# Wideband Direction of Arrival Estimation in the Presence of Unknown Mutual Coupling

**DOI:** 10.3390/s17020230

**Published:** 2017-02-06

**Authors:** Weixing Li, Yue Zhang, Jianzhi Lin, Rui Guo, Zengping Chen

**Affiliations:** School of Electronic Science and Engineering, National University of Defense Technology, Changsha 410073, China; zhangyue05@nudt.edu.cn (Y.Z.); jzlingfkd@163.com (J.L.); guorui11@nudt.edu.cn (R.G.); atrchen@sina.com (Z.C.)

**Keywords:** wideband, uniform linear array, mutual coupling, direction of arrival

## Abstract

This paper investigates a subarray based algorithm for direction of arrival (DOA) estimation of wideband uniform linear array (ULA), under the presence of frequency-dependent mutual coupling effects. Based on the Toeplitz structure of mutual coupling matrices, the whole array is divided into the middle subarray and the auxiliary subarray. Then two-sided correlation transformation is applied to the correlation matrix of the middle subarray instead of the whole array. In this way, the mutual coupling effects can be eliminated. Finally, the multiple signal classification (MUSIC) method is utilized to derive the DOAs. For the condition when the blind angles exist, we refine DOA estimation by using a simple approach based on the frequency-dependent mutual coupling matrixes (MCMs). The proposed method can achieve high estimation accuracy without any calibration sources. It has a low computational complexity because iterative processing is not required. Simulation results validate the effectiveness and feasibility of the proposed algorithm.

## 1. Introduction

Wideband antenna arrays have attracted tremendous interest in various fields including radar, radio astronomy, and wireless communications [1,2]. Most array signal processing algorithms, such as direction of arrival (DOA) and digital beamforming (DBF), are sensitive to array errors because they rely crucially on the prior knowledge of the array manifold. However, actual array systems are inevitably affected by unknown mutual coupling effects between the elements, which may lead to substantial performance degradation [3,4].

A variety of methods have been proposed to mitigate mutual coupling effects in narrowband applications. It is possible to estimate the mutual coupling matrix (MCM) using a network analyzer and computational electromagnetic solvers [5,6]. However, the methods are time-consuming and essentially impractical once arrays are in operation. In [7,8], an alternating minimization procedure for both DOA and mutual coupling parameters was created based on the subspace principle. Procedures of this type, however, usually suffer from serious ambiguous problems and have high computational complexity due to multidimensional searches in nonlinear optimization. An improved method resulted from the application of a group of auxiliary elements on the boundaries of the uniform linear array (ULA), and led to the development of a simple and effective DOA estimation algorithm [9,10,11]. This algorithm requires no calibration sources or iterations and behaves robustly through mutual coupling. A two-dimensional DOA estimation method for an L-shaped sensor array is proposed in [12]. This method employs two collocated antennas to reduce the mutual coupling effects.

In recent years, wideband signals have been widely used in various applications, such as passive radar and sonar, and many wideband DOA estimation algorithms have been proposed [13,14]. Compared to the narrowband situations, DOA estimation for wideband ULA in the presence of unknown mutual coupling seems to be more difficult, since mutual coupling is a frequency-dependent phenomenon [15,16,17]. In [18], the method of moments (MOM) is employed to quantize and eliminate the mutual coupling effects on DOA estimation over a wideband range of frequencies. An improved DOA estimation technique for ultra-wideband electromagnetic waves is studied in [19]. The technique combines interferometry and a modified multiple signal classification (MUSIC) method to mitigate mutual coupling effects. In [20,21], narrowband MCMs at some discrete frequencies are calculated firstly based on the receiving mutual impedance method or the element pattern reconstruction method, then, utilizing the system identification methods, a wideband calibration matrix is obtained, which can be used to compensate the mutual coupling effects at all frequencies. An analysis of wideband DOA estimation taking account of mutual coupling effects is presented in [22,23,24]. The wideband signals are decomposed into multiple discrete narrowband components, and the transforming matrices are decoupled by the MCMs at the corresponding sub-bands to eliminate mutual coupling.

The above wideband DOA estimation algorithms are based on the assumption that the MCMs at all frequencies are perfectly known beforehand. In practice, however, the MCMs vary with environmental factors such as temperature, humidity and vibration, and have to be recalculated when the environment changes. During the operation of array systems, it may be difficult to carry out the measurements owing to heavy workloads.

In this paper, we are concerned with wideband DOA estimation for ULA in the presence of unknown mutual coupling. Based on the property that the MCM at any frequency is a Toeplitz matrix, we propose a new wideband DOA estimation algorithm. The algorithm can estimate the DOAs accurately without any prior knowledge of mutual coupling. It is computationally efficient and feasible for the applications in real-time systems.

This paper is organized as follows: In Section 2, the model of wideband ULA with unknown mutual coupling is presented; Section 3 illustrates the proposed algorithm to estimate wideband DOA without any prior knowledge of mutual coupling; Computer simulations are shown and analyzed in Section 4, followed by conclusions in Section 5.

## 2. Problem Formulation

Consider a wideband ULA with an operating frequency band of $\left[f_{L},f_{H}\right]$. The array is composed of N omnidirectional elements which are equally spaced. The inner-element distance d is defined as the half wavelength of the highest frequency. Assuming that there are M wideband signals $s_{1}\left(t\right),\cdots ,s_{M}\left(t\right)$ from different directions $\theta ={\left[\theta_{1},\cdots ,\theta_{M}\right]}^{T}$ impinging on the array, the output of the ith element can be expressed as:

(1) $$x_{i}\left(t\right)=\sum_{m=1}^{M}s_{m}\left(t-\frac{\left(i-1\right)d\sin\theta_{m}}{c}\right)+n_{i}\left(t\right)$$

where $n_{i}\left(t\right)$ is white noise with a variance of $\sigma^{2}$, and c is the speed of signal propagation. The Fourier transform (FT) of Equation (1) is:

(2) $$X_{i}\left(f\right)=\sum_{m=1}^{M}S_{m}\left(f\right)e^{-j2\pi f\frac{\left(i-1\right)d\sin\theta_{m}}{c}}+N_{i}\left(f\right)$$

Most wideband DOA estimation algorithms divide the full band into a set of frequency bins using discrete Fourier transformation (DFT). Therefore, the array outputs can be written in vector form at $f_{1},\cdots ,f_{K}$:

(3) $$X\left(f_{k}\right)=A(f_{k},\theta )S(f_{k})+N(f_{k})$$

where:

(4) $$\begin{array}{l} X\left(f_{k}\right)={\left[X_{1}\left(f_{k}\right),\cdots ,X_{N}\left(f_{k}\right)\right]}^{T}\\ S\left(f_{k}\right)={\left[S_{1}\left(f_{k}\right),\cdots ,S_{M}\left(f_{k}\right)\right]}^{T}\\ N\left(f_{k}\right)={\left[N_{1}\left(f_{k}\right),\cdots ,N_{N}\left(f_{k}\right)\right]}^{T} \end{array}$$

for $f_{L}\leq f_{k}\leq f_{H}$. $A(f_{k},\theta )\in {\mathbb{C}}^{N\times M}$ is the steering matrix at $f_{k}$, which is defined as:

(5) $$\left\{ \begin{array}{l} A(f_{k},\theta )=\left[a(f_{k},\theta_{1}),\cdots ,a(f_{k},\theta_{M})\right]\\ a(f_{k},\theta_{m})={\left[1,e^{-j2\pi f_{k}d\sin\theta_{m}/c},\cdots ,e^{-j2\pi f_{k}\left(N-1\right)d\sin\theta_{m}/c}\right]}^{T} \end{array}\right.$$

Taking mutual coupling effects into consideration, the received data may be written as:

(6) $$X\left(f_{k}\right)=C\left(f_{k}\right)A(f_{k},\theta )S(f_{k})+N(f_{k})$$

where $C\left(f_{k}\right)\in {\mathbb{C}}^{N\times N}$ is the MCM at $f_{k}$.

It has been shown that $C\left(f_{k}\right)$ can be considered as a banded symmetric matrix in the case of ULA [7]. Indeed, mutual coupling effects tend to be inversely related to the distance between elements and may be negligible for the elements separated by a few wavelengths. Assuming there are P non-zero mutual coupling coefficients, $C\left(f_{k}\right)$ can be determined from:

(7) $$\left\{ \begin{array}{l} C_{i,j}\left(f_{k}\right)=c_{\left|i-j\right|+1}^{\left(k\right)}i,j=1,\cdots ,N\\ 0<|c_{P}^{\left(k\right)}|<\cdots <|c_{2}^{\left(k\right)}|<|c_{1}^{\left(k\right)}|=1\\ c_{i}^{\left(k\right)}=0i>P \end{array}\right.$$

where $c_{i}^{\left(k\right)}$ is the mutual coupling coefficient between the first and the ith element at $f_{k}$. The number of non-zero mutual coupling coefficients can be obtained by initial measurements using network analyzers. Since mutual coupling varies slowly, these measurements may be carried out only once.

As can be seen from Equation (6), wideband array outputs are affected by the MCMs at all frequencies in the band. If the mutual coupling is unknown, $C\left(f_{k}\right)$ in Equation (6) cannot be obtained. In order to compensate Equation (6), measurements using a network analyzer must be carried out to calculate mutual coupling coefficients. However, this method is time-consuming. In this paper, however, we are concerned with estimating wideband DOA when mutual coupling is unknown.

## 3. Subarray-Based Wideband DOA Estimation Algorithm

### 3.1. Derivation of Focusing Matrices

A representative solution to wideband DOA estimation is the coherent subspace method (CSM) [13]. In CSM, the correlation matrices at different frequency bins are aligned by a series of focusing matrices to form a narrowband subspace and, subsequently, the MUSIC method can be applied. In this section, we construct the focusing matrices in the presence of unknown mutual coupling.

Considering the special structure of MCM in Equation (7), we take P−1 elements on each side of the array as auxiliary elements to combat mutual coupling effects. Define a m×k selecting matrix as:

(8) $$F_{m,n}^{k}=\left[0_{m\times n},\;I_{m},\;0_{m\times \left(k-m-n\right)}\right]$$

where $0_{m\times n}$ is a m×n zero matrix, and $I_{m}$ is the m×m identity matrix.

The outputs of the remaining N−2P+2 elements, denoted as the middle subarray, can be selected from the whole array outputs:

(9) $$\begin{array}{cc} \tilde{X}(f_{k}) & =F_{N_{0},P-1}^{N}X\left(f_{k}\right)\\ & =\tilde{C}(f_{k})A(f_{k})S(f_{k})+\tilde{N}(f_{k}) \end{array}$$

where $N_{0}=N-2P+2$, and $\tilde{N}(f_{k})=F_{N_{0},P-1}^{N}N(f_{k})$ is a $N_{0}\times 1$ noise vector. $\tilde{C}(f_{k})=F_{N_{0},P-1}^{N}C(f_{k})$ denotes the MCM of the middle subarray at $f_{k}$, which can be written in the form of:

(10) $$\tilde{C}(f_{k})=\left[\begin{array}{ccccccccccc} c_{P}^{\left(k\right)} & \cdots & c_{2}^{\left(k\right)} & 1 & c_{2}^{\left(k\right)} & \cdots & c_{P}^{\left(k\right)} & 0 & \cdots & \cdots & 0\\ 0 & c_{P}^{\left(k\right)} & \cdots & c_{2}^{\left(k\right)} & 1 & c_{2}^{\left(k\right)} & \cdots & c_{P}^{\left(k\right)} & 0 & \cdots & 0\\ \vdots & \vdots & \vdots & \vdots & \ddots & \ddots & \ddots & \ddots & \ddots & \vdots & \vdots \\ 0 & \cdots & \cdots & 0 & c_{P}^{\left(k\right)} & \cdots & c_{2}^{\left(k\right)} & 1 & c_{2}^{\left(k\right)} & \cdots & c_{P}^{\left(k\right)} \end{array}\right]$$

Here we define ${\tilde{c}}_{k}={\left[c_{P}^{\left(k\right)},\cdots ,c_{2}^{\left(k\right)},1,c_{2}^{\left(k\right)},\cdots ,c_{P}^{\left(k\right)}\right]}^{T}\in {\mathbb{C}}^{\left(2P-1\right)\times 1}$ as the mutual coupling vector at $f_{k}$.

As shown in Equation (10), the non-zero mutual coupling coefficients of each element in the middle subarray have the same values, differing only in positions. Making use of this property, we construct the focusing matrices by the middle array outputs instead of the whole array outputs. The intention, therefore, is to eliminate the influence of mutual coupling on wideband DOA estimation.

Without loss of generality, we define $f_{0}\in \left[f_{L},f_{H}\right]$ as the focusing frequency. The correlation matrix of the middle array outputs at $f_{0}$ is given by:

(11) $$\begin{array}{cc} \tilde{R}\left(f_{0}\right) & =E\left[\tilde{X}(f_{0}){\tilde{X}}^{H}(f_{0})\right]\\ & =\tilde{C}\left(f_{0}\right)A\left(f_{0},\theta \right)R_{s}\left(f_{0}\right)A^{H}\left(f_{0},\theta \right){\tilde{C}}^{H}\left(f_{0}\right)+\sigma_{0}^{2}I_{N_{0}} \end{array}$$

where E[ · ] denotes the expectation operator, and $\sigma_{0}^{2}$ is the power of noise at $f_{0}$. The superscript H denotes the conjugate transpose. $R_{s}\left(f_{0}\right)=E\left[S\left(f_{0}\right)S^{H}\left(f_{0}\right)\right]$ is the correlation matrix of incident signals at $f_{0}$.

Due to the special structure of $\tilde{C}\left(f_{0}\right)$ in Equation (10), for the signal from $\theta_{m}$, we have:

(12) $$\begin{array}{cc} \tilde{C}\left(f_{0}\right)a\left(f_{0},\theta_{m}\right) & =\left[\begin{array}{c} c_{P}^{\left(0\right)}+c_{P-1}^{\left(0\right)}\beta_{m}+\cdots +c_{2}^{\left(0\right)}\beta_{m}^{P-2}+\beta_{m}^{P-1}+c_{2}^{\left(0\right)}\beta_{m}^{P}+\cdots +c_{P}^{\left(0\right)}\beta_{m}^{2P-2}\\ \beta_{m}\left(c_{P}^{\left(0\right)}+c_{P-1}^{\left(0\right)}\beta_{m}+\cdots +c_{2}^{\left(0\right)}\beta_{m}^{P-2}+\beta_{m}^{P-1}+c_{2}^{\left(0\right)}\beta_{m}^{P}+\cdots +c_{P}^{\left(0\right)}\beta_{m}^{2P-2}\right)\\ \vdots \\ \beta_{m}^{N_{0}-1}\left(c_{P}^{\left(0\right)}+c_{P-1}^{\left(0\right)}\beta_{m}+\cdots +c_{2}^{\left(0\right)}\beta_{m}^{P-2}+\beta_{m}^{P-1}+c_{2}^{\left(0\right)}\beta_{m}^{P}+\cdots +c_{P}^{\left(0\right)}\beta_{m}^{2P-2}\right) \end{array}\right]\\ & =g\left(f_{0},{\tilde{c}}_{0},\beta_{m}\right)\tilde{a}\left(f_{0},\theta_{m}\right) \end{array}$$

where $\beta_{m}=e^{-j2\pi f_{0}d\sin\theta_{m}/c}$. ${\tilde{c}}_{0}$ is the mutual coupling vector at $f_{0}$. $g\left(f_{0},{\tilde{c}}_{0},\theta_{m}\right)=c_{P}^{\left(0\right)}+c_{P-1}^{\left(0\right)}\beta_{m}+\cdots +c_{2}^{\left(0\right)}\beta_{m}^{P-2}+\beta_{m}^{P-1}+c_{2}^{\left(0\right)}\beta_{m}^{P}+\cdots +c_{P}^{\left(0\right)}\beta_{m}^{2P-2}$ is a function of the mutual coupling coefficients, frequency and DOA. $\tilde{a}\left(f_{0},\theta_{m}\right)\in {\mathbb{C}}^{N_{0}\times 1}$ is the ideal steering vector of the middle subarray defined as:

(13) $$\tilde{a}\left(f_{0},\theta_{m}\right)={\left[1,\beta_{m},\cdots ,\beta_{m}^{N_{0}-1}\right]}^{T}$$

From Equation (12), we can obtain:

(14) $$\begin{array}{cc} \tilde{C}\left(f_{0}\right)A\left(f_{0},\theta \right) & =[g\left(f_{0},{\tilde{c}}_{0},\theta_{1}\right)\tilde{a}\left(f_{0},\theta_{1}\right),\cdots ,g\left(f_{0},{\tilde{c}}_{0},\theta_{M}\right)\tilde{a}\left(f_{0},\theta_{M}\right)]\\ & =\tilde{A}\left(f_{0},\theta \right)D\left(f_{0},{\tilde{c}}_{0},\theta \right) \end{array}$$

where

(15) $$\begin{array}{l} \tilde{A}\left(f_{0},\theta \right)=\left[\tilde{a}\left(f_{0},\theta_{1}\right),\cdots ,\tilde{a}\left(f_{0},\theta_{M}\right)\right]\\ D\left(f_{0},{\tilde{c}}_{0},\theta \right)=diag\left\{g\left(f_{0},{\tilde{c}}_{0},\theta_{1}\right),\cdots ,g\left(f_{0},{\tilde{c}}_{0},\theta_{M}\right)\right\} \end{array}$$

For simplicity of notation, we suppress the DOA variable and represent $A(f_{0},\theta )$ by $A(f_{0})$ and $D\left(f_{0},{\tilde{c}}_{0},\theta \right)$ by $D\left(f_{0},{\tilde{c}}_{0}\right)$.

By Substituting Equation (14) into Equation (11) and subtracting the noise-power matrix, we can obtain the focusing noise-free correlation matrix at $f_{0}$:

(16) $$\tilde{P}\left(f_{0}\right)=\tilde{A}\left(f_{0}\right)D\left(f_{0},{\tilde{c}}_{0}\right)R_{s}\left(f_{0}\right)D^{H}\left(f_{0},{\tilde{c}}_{0}\right){\tilde{A}}^{H}\left(f_{0}\right)$$

In the same way, we can derive the noise-free correlation matrix at each frequency bin as $\tilde{P}\left(f_{k}\right)=\tilde{R}\left(f_{k}\right)-\sigma_{k}^{2}I_{N_{0}}$, where $\tilde{R}\left(f_{k}\right)=E\left[\tilde{X}(f_{k}){\tilde{X}}^{H}(f_{k})\right]$. $\sigma_{k}^{2}$ denotes the noise power at $f_{k}$, which can be estimated by:

(17) $$\sigma_{k}^{2}=\frac{1}{N_{0}-M}\sum_{i=M+1}^{N_{0}}\lambda_{i}\left(\tilde{R}\left(f_{k}\right)\right)$$

where $\lambda_{i}\left(\tilde{R}\left(f_{k}\right)\right)i=M+1,\cdots ,N_{0}$ denotes the small eigenvalues of $\tilde{R}\left(f_{k}\right)$.

Here we aim at transforming the noise-free correlation matrix at each frequency bin to $\tilde{P}\left(f_{0}\right)$. Based on the two-sided correlation transformation (TCT) criterion [13], the focusing matrix is selected to satisfy Equation (18):

(18) $$T\left(f_{k}\right)\tilde{P}\left(f_{k}\right)T^{H}\left(f_{k}\right)=\tilde{P}\left(f_{0}\right)$$

Generally, the focusing matrix is constrained to a unitary matrix. Therefore, it can be determined by solving the following minimization problem:

(19) $$\begin{array}{l} \min_{T\left(f_{k}\right)}{\left\|\tilde{P}\left(f_{0}\right)-T\left(f_{k}\right)\tilde{P}\left(f_{k}\right)T^{H}\left(f_{k}\right)\right\|}_{F}\\ \mathrm{subject}\;\mathrm{to}\;T\left(f_{k}\right)T^{H}\left(f_{k}\right)=I_{N_{0}} \end{array}$$

for k=1,2,⋯,K, where ${\left\|\;\cdot \;\right\|}_{F}$ denotes the Frobenius norm of a matrix.

It has been shown in [13] that the optimal solution of Equation (19) is given by:

(20) $$T\left(f_{k}\right)=U\left(f_{0}\right)U^{H}\left(f_{k}\right)$$

where $U\left(f_{0}\right)$ and $U\left(f_{k}\right)$ are the eigenvector matrices of $\tilde{P}\left(f_{0}\right)$ and $\tilde{P}\left(f_{k}\right)$, respectively.

Using Equation (20), we can construct a focusing matrix corresponding to the correlation matrix at each frequency bin. Therefore, the general focused correlation matrix is obtained as:

(21) $$P=\frac{1}{K}\sum_{k=1}^{K}T\left(f_{k}\right)\tilde{P}\left(f_{k}\right)T^{H}\left(f_{k}\right)$$

The selection of focusing frequency in Equation (19) is based on the criterion that minimizes the fitting error, which is a function of $f_{0}$. Details of the derivation of the optimal $f_{0}$ are shown in [13]. Here we only give the result:

(22) $$f_{0}=\arg\;\min_{f_{0}}{\sum_{i=1}^{M}\left|\sigma_{i}\left(\tilde{P}\left(f_{0}\right)\right)-\frac{\mu_{i}}{K}\right|}^{2}$$

where $\mu_{i}=\sum_{k=1}^{K}\sigma_{i}\left(\tilde{P}\left(f_{k}\right)\right)$. $\sigma_{i}\left(\tilde{P}\left(f_{k}\right)\right)$ denotes the singular values of $\tilde{P}\left(f_{k}\right)$. M is the number of incident signals, and K is the number of frequency bins. By searching $f_{k}\left(k=1,2,\cdots ,K\right)$ in Equation (22), the optimal focusing frequency $f_{0}$ can be found.

If we directly deal with $f_{k}\left(k=1,2,\cdots ,K\right)$, narrowband DOA methods, such as MUSIC, should be performed on all of the frequency bins. This kind of wideband DOA method is time-consuming. In this manuscript, the correlation matrices at different frequency bins are aligned by a series of transforming matrices. Therefore, the MUSIC method can be performed only at the focusing frequency bin, which is computationally efficient. Moreover, these transforming matrices contain the information at each frequency bin $f_{k}\left(k=1,2,\cdots ,K\right)$. As a result, the DOA estimation can also reach high accuracy.

### 3.2. DOA Estimation with Unknown Mutual Coupling

We have constructed the focusing matrices using the middle array outputs, where the energy of wideband signals are condensed at the focusing frequency $f_{0}$. Subsequently, wideband DOA estimation problems can be addressed in narrowband.

In an ideal case, the noise-free correlation matrices at all frequency bins are transformed to the focusing noise-free correlation matrix. Substituting Equations (16) and (18) into Equation (21) yields:

(23) $$P=\tilde{A}\left(f_{0}\right)R_{s}^{\prime }\left(f_{0},{\tilde{c}}_{0}\right){\tilde{A}}^{H}\left(f_{0}\right)$$

where $R_{s}^{\prime }\left(f_{0},{\tilde{c}}_{0}\right)=D\left(f_{0},{\tilde{c}}_{0}\right)R_{s}\left(f_{0}\right)D^{H}\left(f_{0},{\tilde{c}}_{0}\right)$.

On the assumption that $g\left(f_{0},{\tilde{c}}_{0},\theta_{m}\right)\neq 0$ for m=1,⋯,M (the situations where $g\left(f_{0},{\tilde{c}}_{0},\theta_{m}\right)=0$ will be discussed further), $D\left(f_{0},{\tilde{c}}_{0}\right)$ is of full rank. Owing to the diagonal structure of $D\left(f_{0},{\tilde{c}}_{0}\right)$, $R_{s}^{\prime }\left(f_{0},{\tilde{c}}_{0}\right)$ has the same rank as $R_{s}\left(f_{0}\right)$. Therefore, the general focused correlation matrix in Equation (23) may be eigen-decomposed as:

(24) $$P=E_{s}\Lambda_{s}E_{s}^{H}+E_{n}\Lambda_{n}E_{n}^{H}$$

where $E_{s}$ and $E_{n}$ represent the signal subspace and the noise subspace, respectively. $\Lambda_{s}$ is a diagonal matrix formed by the M principle eigenvalues, and $\Lambda_{n}$ is formed from the remaining $N_{0}-M$ eigenvalues. Most DOA estimation methods, such as MUSIC, CSM, and ISM, require that the number of incident signals is known. The number of principal eigenvalues equals to the number of incident signals.

It can be seen from Equation (23) that the unknown mutual coupling coefficients are completely contained in $R_{s}^{\prime }\left(f_{0},{\tilde{c}}_{0}\right)$. Therefore, they do not affect the orthogonality between the noise space and the steering matrix of the middle subarray. According to the subspace principle, we have:

(25) $$span\left\{\tilde{A}\left(f_{0}\right)\right\}=span\left\{E_{s}\right\}⊥span\left\{E_{n}\right\}$$

As a result, it is possible to employ the MUSIC method without mutual coupling compensation:

(26) $$P_{MUSIC}\left(\theta \right)=\frac{1}{{\left\|E_{n}^{H}\tilde{a}\left(f_{0},\theta \right)\right\|}_{F}^{2}}$$

By searching the peaks of $P_{MUSIC}\left(\theta \right)$, the DOA estimation of incident signals can be obtained.

### 3.3. Discussion

In Section 3.2, we assume that $g\left(f_{0},{\tilde{c}}_{0},\theta_{m}\right)\neq 0$ for m=1,⋯,M. However, in some particular conditions, there may exist ϑ∈[−π/2,π/2] satisfying $g\left(f_{0},{\tilde{c}}_{0},\vartheta \right)=0$. In this case, signals from these angles will be lost. These angles, the so called blind angles, are caused by mutual coupling effects [10], and in the case of narrowband arrays, they are difficult to cope with.

In wideband applications, if the mutual coupling coefficients at the focusing frequency satisfy $g\left(f_{0},{\tilde{c}}_{0},\vartheta \right)=0$, signals from ϑ will be missed. In order to address this problem, we select a different frequency $f_{0}^{\prime }$ as the focusing frequency, and the general focused correlation matrix can be obtained as:

(27) $$P^{\prime }=\tilde{A}\left(f_{0}^{\prime }\right)D\left(f_{0}^{\prime },{\tilde{c}}_{0}^{\prime }\right)R_{s}\left(f_{0}^{\prime }\right)D^{H}\left(f_{0}^{\prime },{\tilde{c}}_{0}^{\prime }\right){\tilde{A}}^{H}\left(f_{0}^{\prime }\right)$$

where ${\tilde{c}}_{0}^{\prime }$ denotes the mutual coupling vector at $f_{0}^{\prime }$. The optimal focusing frequency can be obtained by searching minimum value of Equation (22). Therefore, we can sort the values of Equation (22) in ascending order, and select $f_{0}^{\prime }$ corresponding to the second smallest values as another focusing frequency.

Since g(f,c,θ) is a function of the mutual coupling coefficients which change with the frequency, it rarely happens that both $g\left(f_{0},{\tilde{c}}_{0},\vartheta \right)$ and $g\left(f_{0}^{\prime },{\tilde{c}}_{0}^{\prime },\vartheta \right)$ are equal to zero, so $D\left(f_{0}^{\prime },{\tilde{c}}_{0}^{\prime }\right)$ is of full rank. Therefore, ϑ can be estimated using $P^{\prime }$.

In practice, it is difficult to select a proper focusing frequency to avoid the blind angles without any prior knowledge of mutual coupling. However, we can choose several different frequencies $f_{0}^{\left(1\right)},\cdots ,f_{0}^{\left(K^{\prime }\right)}$ as the focusing frequencies and, subsequently, the corresponding spatial spectra $P_{MUSIC}^{\left(1\right)},\cdots ,P_{MUSIC}^{\left(K^{\prime }\right)}$ in Equation (26) can be obtained. It seems that blind angles may not exist at all of the focusing frequencies owing to frequency-dependent mutual coupling coefficients. Therefore, to avoid blind angles, we can calculate the average spatial spectrum as follows:

(28) $${\bar{P}}_{MUSIC}=\frac{1}{K^{\prime }}\left(P_{MUSIC}^{\left(1\right)}+\cdots +P_{MUSIC}^{\left(K^{\prime }\right)}\right)$$

Finally, the complete descriptions of the proposed algorithm are summarized as follows:

(1) Divide the array outputs into non-overlapping time slices, each containing K samples, and apply DFT in each slice to sample the spectrum of the outputs at a group of frequency bins $f_{k}\;\left(k=1,\cdots ,K\right)$.
(2) Select the outputs of the middle subarray from Equation (9). The number of nonzero mutual coupling coefficients can be obtained by preliminary measurements at the lowest operating frequency.
(3) Find $\tilde{P}\left(f_{0}\right)$ at the preselected focusing frequency. For each frequency bin, determine $\tilde{P}\left(f_{k}\right)$ and construct the focusing matrix $T\left(f_{k}\right)$ using Equation (20).
(4) Calculate the general focused correlation matrix P using Equation (21).
(5) Apply the MUSIC method to P for the estimation of the DOA.
(6) In order to avoid blind angles, we choose several focusing frequencies and repeat steps (3) to (5) and then search the peaks of the averaging spectrums in Equation (28). However, considering the cost of computation, this step is usually skipped unless we find some signals are lost.

## 4. Simulation Results

In this section, several representative simulations are carried out to demonstrate the performance of the proposed algorithm.

**Figure 1:**
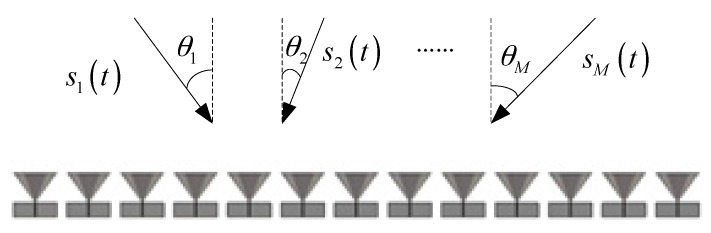
Setup of the array antenna.

Consider a wideband monopole antenna array with N=13 elements arranged uniformly in a line, which is shown in Figure 1. The operation frequency band is B=500 MHz with a center frequency of $f_{c}=1.5\;\mathrm{GHz}$. With the purpose of avoiding grating lobes, the distance between neighboring elements is set to d=0.086 m, which is the half wavelength of the highest frequency. The wideband mutual coupling coefficients are generated by the computer simulation technology Microwave Studio (CST MW) software. In this paper, we assume that gain and phase errors have already been compensated [25], and only mutual coupling errors are concerned.

**Figure 2:**
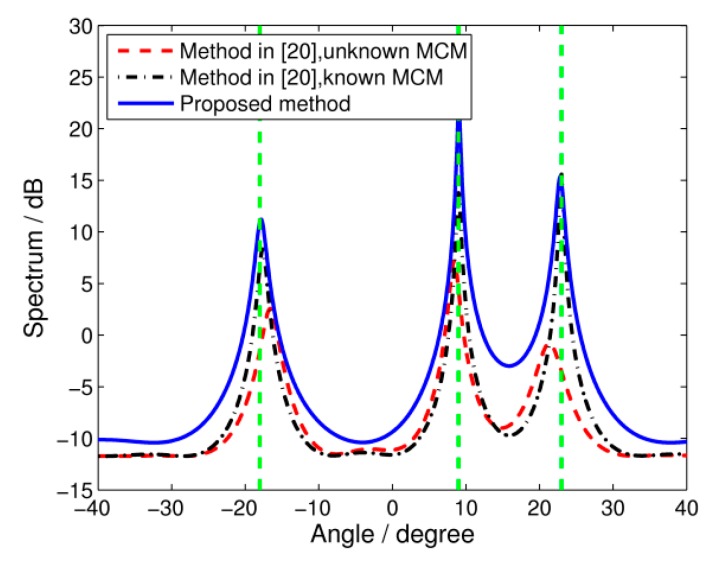
Spatial spectrums of wideband DOA estimation. SNR = 5 dB, snapshots = 100.

In the first simulation, three incoherent signals, with a bandwidth of 500 MHz covering [1.25 GHz, 1.75 GHz], impinge on the array from −18°, 9°, and 23°. The signal to noise ratio (SNR) is set to 5 dB and 100 snapshots are collected at each frequency bin. Two elements on both sides are treated as auxiliary elements, and the other *N*_0_ = 9 elements are treated as the middle array. The spatial spectrum of DOA estimation using the proposed algorithm is shown in Figure 2. In comparison, we also illustrate the results using the algorithm in [20], which employ the system identification method to decouple mutual coupling effects. Here we assume that the MCMs at all frequency bins are known precisely. However, such information may be unavailable in practice. Therefore, the results of the traditional TCT algorithm in [13] with unknown mutual coupling are also given. It is shown in the figure that both the proposed algorithm and the algorithm from [20] with known MCMs are able to estimate the DOAs successfully since the curves of their spatial spectrum share sharp peaks at the correct directions. However, without prior knowledge of the MCMs, the TCT algorithm yields poor resolution and the curve peaks deviate from their true positions.

The second simulation is carried out to evaluate the precision of DOA estimation, which may be measured by the root mean-squared error (RMSE) defined as:

(29) $$\theta_{rmse}=\sqrt{\sum_{l=1}^{L}\sum_{i=1}^{M}{\left({\hat{\theta }}_{i}\left(l\right)-\theta_{i}\right)}^{2}/\left(ML\right)}$$

where L and M denote the number of incident signals and the Monte Carlo trials, respectively. ${\hat{\theta }}_{i}\left(l\right)$ is the estimation of $\theta_{i}$ in the lth trial. Here we ran 200 Monte Carlo trials for each condition in the simulations unless specifically mentioned.

**Figure 3:**
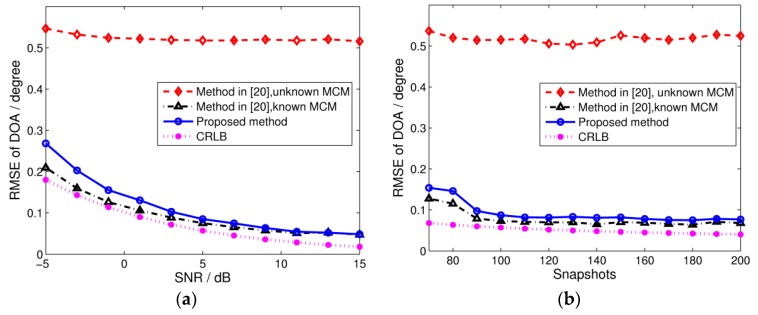
RMSE of wideband DOA estimation with 200 Monte Carlo trials. (**a**) RMSE versus input SNR, where snapshots = 100; and (**b**) RMSE versus the number of snapshots, where SNR = 5 dB.

The incident angles are the same as in Simulation 1. The RMSE of wideband DOA estimation against the input SNR and the number of snapshots at each frequency bin are shown in Figure 3a,b, respectively. The Cramer-Rao lower bound (CRLB) of the DOA estimation in the presence of mutual coupling (computed as shown in the Appendix A) is also illustrated. As we can see, the performance of the TCT algorithm is seriously degraded in the presence of unknown mutual coupling, and the RMSE can’t be improved by increasing the input SNR or the number of snapshots. In contrast, the unknown mutual coupling has little influence on the proposed algorithm, which achieves considerable accuracy. Since the proposed algorithm loses some array apertures in order to eliminate mutual coupling effects, the performance is slightly worse than the algorithm in [20] with known MCMs at low SNR. However, it still reaches high estimation precision and is close to the case without mutual coupling at high SNR. Furthermore, there is no need to calculate any MCMs, so the proposed algorithm has low computational requirements and achieves high real-time performance.

**Figure 4:**
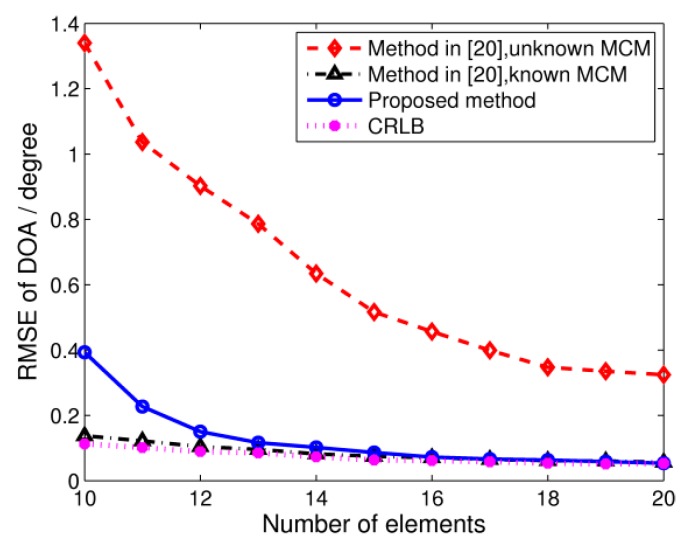
RMSE of DOA estimation versus the number of array elements. SNR = 5 dB, snapshots = 100.

Setting the SNR to 5 dB and the number of snapshots to 100, the effect of the array size is illustrated in Figure 4. As is evident from the figure, the proposed algorithm outperforms the TCT algorithm with unknown MCM. When array elements are not sufficient, the performance of our algorithm deteriorates because only the elements from the middle subarray are used. In spite of this, the RMSE of our algorithm is almost the same as the algorithm from [20] with known MCMs in the case of a large array size.

**Figure 5:**
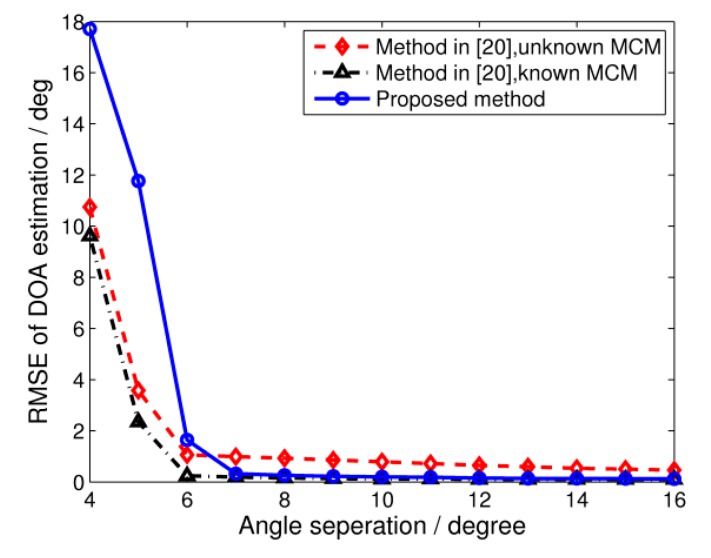
RMSE of DOA estimation versus the angle separation of incident signals. SNR = 0 dB, snapshots = 64.

The resolution of the DOA estimation is investigated in the third simulation. Keeping other conditions the same as in Simulation 1, we consider two wideband signals impinging on the array. The direction of one signal is fixed at −25°, while that of the other signal varies from −21° to −9°. The RMSE of the DOA estimation against angle separation is shown in Figure 5. It is seen that the proposed algorithm behaves robustly in the presence of unknown MCMs, and the RMSE is smaller than 0.3° when the two signals are separated by 8°.

**Figure 6:**
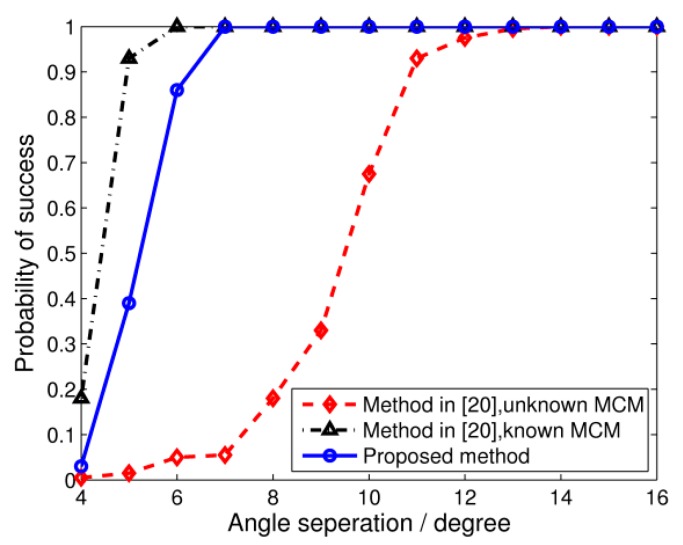
Success probability of DOA estimation versus the angle separation of incident signals. SNR = 0 dB, snapshots = 64.

Figure 6 depicts the success probabilities which are derived from 500 trials. We assume that the two signals are successfully resolved if both DOA estimation bias are smaller than 1°, i.e., $\max\{|{\hat{\theta }}_{1}-\theta_{1}|,|{\hat{\theta }}_{2}-\theta_{2}|\}<1$, where ${\hat{\theta }}_{i}\;\left(i=1,2\right)$ stands for the estimation of $\theta_{i}$. As can be seen from the figure, with known MCMs, the signals can be well resolved with an angle separation of 6°. The proposed algorithm, with slight resolution performance deterioration, still has the potential to reach 100% success probabilities for signals separated by 8°. However, the TCT algorithm with unknown MCMs is not able to resolve the two signals unless they are separated by more than 13°.

**Figure 7:**
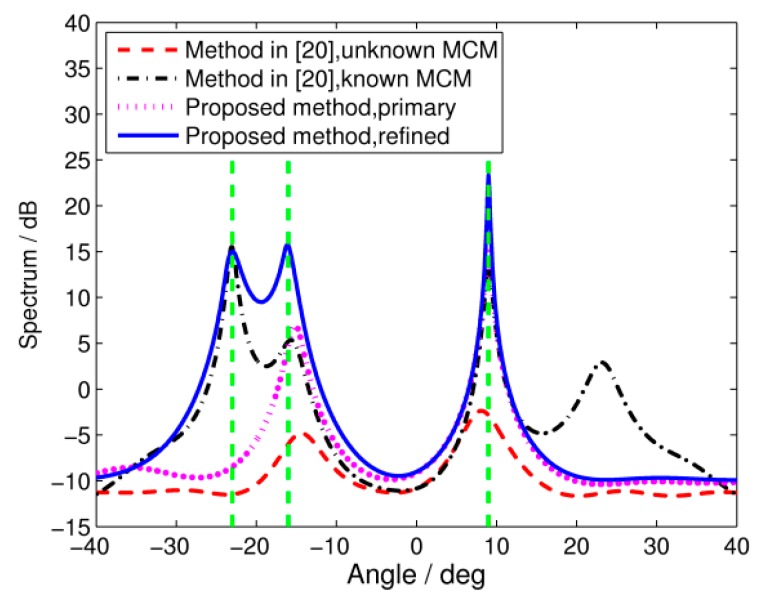
Spatial spectrums of wideband DOA estimation with blind angles. SNR = 5 dB, snapshots = 100.

In the fourth simulation, we consider the scenario where blind angles exist. We generate a group of mutual coupling coefficients which satisfy $g\left(f_{0},{\tilde{c}}_{0},\theta_{3}\right)\approx 0$ when $\theta_{3}=\pm 23^{\circ }$, $f_{0}=1.5\;\mathrm{GHz}$. According to the analysis in Section 3.3, signals from ±23° will be missed if we choose 1.5 GHz as the focusing frequency. The incident signals are assumed to be the same as Simulation 1. As illustrated from the spatial spectrums in Figure 7, both the primary proposed algorithm and the TCT algorithm with unknown MCMs miss the signal from $\theta_{3}=23^{\circ }$. With known MCMs, the algorithm from [20] can successfully derive the blind angle of 23°. However, the DOA estimation is still affected by mutual coupling, since a pseudo-peak appears at the other blind angle of −23°. The result is similar to the conclusion from [10]. In order to cope with this problem, we carried out the refined algorithm presented in Section 3.3 by calculating the mean value of spectrums derived at the focusing frequencies of 1.4 GHz, 1.5 GHz, and 1.6 GHz. The results show that all the signals can be resolved accurately without any pseudo-peak.

## 5. Conclusions

In this paper, we present a wideband DOA estimation algorithm for ULA in the presence of unknown mutual coupling. Based on the special structure of the MCM, focusing matrices are constructed using the outputs of the middle subarray to eliminate mutual coupling effects. The algorithm can estimate the DOA accurately without any prior information of mutual coupling. It does not require any iterative procedures and has low computational requirements. Moreover, an improved approach is proposed to cope with the blind angles. The effectiveness of the proposed algorithm is verified by the simulations.

## Appendix A

The CRLB of the wideband DOA estimation is derived as follows:

Suppose the frequency band is divided into K frequency bins, the received data in the presence of mutual coupling can be expressed by Equation (A1):

(A1) $$X\left(f_{k}\right)=C\left(f_{k}\right)A(f_{k},\theta )S(f_{k})+N(f_{k})\;\mathrm{for}\;k=1,2,\cdots ,K$$

where $N(f_{k})$ is white noise with a variance of $K\sigma^{2}$.

Defining $Y\left(f_{k}\right)=C\left(f_{k}\right)A(f_{k},\theta )S(f_{k})$, we can construct the stack vectors as:

(A2) X=Y+N

where:

(A3) $$\left\{ \begin{array}{l} X={\left[X^{T}\left(f_{1}\right),\cdots ,X^{T}\left(f_{K}\right)\right]}^{T}\in {\mathbb{C}}^{NK\times 1}\\ Y={\left[Y^{T}\left(f_{1}\right),\cdots ,Y^{T}\left(f_{K}\right)\right]}^{T}\in {\mathbb{C}}^{NK\times 1}\\ N={\left[N^{T}\left(f_{1}\right),\cdots ,N^{T}\left(f_{K}\right)\right]}^{T}\in {\mathbb{C}}^{NK\times 1} \end{array}\right.$$

The logarithm of the probability density (PDF) for J statistically-independent observations can be written as [25]:

(A4) $$L\left(\theta \right)=\mathrm{const}-J{\left(X-Y\right)}^{H}R_{N}^{-1}\left(X-Y\right)$$

where $R_{N}=E\left\{N^{H}N\right\}=K\sigma^{2}I_{NK}$.

The first order derivative with respect to $\theta_{i}\left(i=1,2,\cdots ,M\right)$ is:

(A5) ∂L∂θi=2JRe{∂YH∂θiRN−1(X−Y)}

The second order derivative is given by:

(A6) ∂2L∂θiθj=2JRe{∂YH∂θiθjRN−1(X−Y)−∂YH∂θiRN−1∂Y∂θj}=−2JRe{∂YH∂θiRN−1∂Y∂θj}

Therefore, we can obtain the Fisher matrix:

(A7) Fi,j=−∂2L∂θiθj=2JRe{∂YH∂θiRN−1∂Y∂θj}

Equation (A7) can be written in the form of:

(A8) F=2JKσ2Re{HHH}

where:

(A9) $$H=\frac{\partial Y}{\partial \theta^{T}}=\left[\frac{\partial Y}{\partial \theta_{1}},\frac{\partial Y}{\partial \theta_{2}},\cdots ,\frac{\partial Y}{\partial \theta_{P}}\right]\in {\mathbb{C}}^{NK\times P}$$

For any i∈[1,M], we have:

(A10) $$\frac{\partial Y}{\partial \theta_{i}}={\left[\frac{\partial Y^{T}\left(f_{1}\right)}{\partial \theta_{i}},\frac{\partial Y^{T}\left(f_{2}\right)}{\partial \theta_{i}},\cdots ,\frac{\partial Y^{T}\left(f_{K}\right)}{\partial \theta_{i}}\right]}^{T}$$

where:

(A11) $$\frac{\partial Y\left(f_{k}\right)}{\partial \theta_{j}}=C\left(f_{k}\right)\frac{\partial A\left(\theta ,f_{k}\right)}{\partial \theta_{j}}s\left(f_{k}\right)$$

Substituting Equations (A9)–(A11) into Equation (A8), the Fisher matrix can be calculated. Subsequently, we can obtain the CRLB of the wideband DOA estimation in the presence of mutual coupling.
